# Caffeine extends life span, improves healthspan, and delays age-associated pathology in *Caenorhabditis elegans*

**DOI:** 10.1186/2046-2395-1-9

**Published:** 2012-12-01

**Authors:** George L Sutphin, Emma Bishop, Melana E Yanos, Richard M Moller, Matt Kaeberlein

**Affiliations:** 1 Department of Pathology, University of Washington, Box 357470, Seattle, 98195-7470, WA, USA; 2 Molecular and Cellular Biology Program, University of Washington, Box 357275, Seattle, 98195-7275, WA, USA; 3 Department of Psychology, University of Washington, Box 351525, Seattle, 98195-1525, WA, USA; 4 Institute of Aging Research, Guangdong Medical College, Dongguan, 523808, China

**Keywords:** Aging, Life span, Longevity, Healthspan, Worms, Caffeine, Proteotoxicity, Neurodegeneration

## Abstract

**Background:**

The longevity of an organism is influenced by both genetic and environmental factors. With respect to genetic factors, a significant effort is being made to identify pharmacological agents that extend life span by targeting pathways with a defined role in the aging process. On the environmental side, the molecular mechanisms responsible for the positive influence of interventions such as dietary restriction are being explored. The environment experienced by humans in modern societies already contains countless compounds that may influence longevity. Understanding the role played by common compounds that substantially affect the aging process will be critical for predicting and interpreting the outcome of introducing new interventions. Caffeine is the most widely used psychoactive drug worldwide. Prior studies in flies, worms, and mice indicate that caffeine may positively impact age-associated neurodegenerative pathology, such as that observed in Alzheimer’s disease.

**Results:**

Here we report that caffeine is capable of extending life span and improving healthspan in *Caenorhabditis elegans*, a finding that is in agreement with a recently published screen looking for FDA-approved compounds capable of extending worm life span. Life span extension using caffeine displays epistatic interaction with two known longevity interventions: dietary restriction and reduced insulin signaling. Caffeine treatment also delays pathology in a nematode model of polyglutamine disease.

**Conclusions:**

The identification of caffeine as a relevant factor in aging and healthspan in worms, combined with prior work in both humans and rodents linking caffeine consumption to reduced risk of age-associated disease, suggests that caffeine may target conserved longevity pathways. Further, it may be important to consider caffeine consumption when developing clinical interventions, particularly those designed to mimic dietary restriction or modulate insulin/IGF-1-like signaling. The positive impact of caffeine on a worm model of polyglutamine disease suggests that chronic caffeine consumption may generally enhance resistance to proteotoxic stress and may be relevant to assessing risk and developing treatments for human diseases like Alzheimer’s and Huntington’s disease. Future work addressing the relevant targets of caffeine in models of aging and healthspan will help to clarify the underlying mechanisms and potentially identify new molecular targets for disease intervention.

## Background

Numerous interventions have been identified that extend life span across an evolutionarily diverse range of organisms [1,2]. These include external (environmental) interventions, such as dietary restriction, heat shock, or treatment with a pharmacological agent, as well as internal (genetic) interventions, such as reduced target of rapamycin (TOR) signaling or reduced insulin/IGF-1-like signaling (IIS). In most cases, studies identifying these interventions are carried out using a genetically homogenous population in a controlled, low-risk, and pathogen-free environment. Interventions that are successful under laboratory conditions are beginning to be introduced into human clinical trials. As these trials progress, it will be important to understand how the artificial nature of the populations and environments in the laboratory setting may impact the outcomes of specific interventions when applied under more variable conditions. In terms of genetics, efforts are underway to understand the effects of dietary restriction in genetically heterogeneous populations. Early evidence indicates that the benefits observed in laboratory populations might not be universally realized by all members of genetically diverse populations [3-7]. From an environmental perspective, human populations are exposed to a wide range of diets, climates, and pharmacological agents that are not present in the laboratory setting. Understanding the impact of these factors on longevity and age-associated disease will be important for predicting unintended effects that might arise from introducing novel interventions.

Caffeine is the most widely used psychoactive substance worldwide. Average consumption in the US is 168 mg/person/day (equivalent to 1 to 2 cups of Starbucks® coffee) and reaches 414 mg/person/day in the Netherlands [8]. Chronic, moderate consumption of caffeine has been linked with reduced risk of age-associated neurodegenerative disorders in humans, including dementia [9], Alzheimer’s disease [9-11], and Parkinson’s disease [12-15]. In addition, studies performed on elderly human populations have correlated habitual caffeine consumption with reduced mortality [16,17] and improvements in various measures of healthspan, including reduced cognitive decline [18,19], improved memory [20-22], and increased motor speed [20]. While other studies failed to find similar correlations, the differences are attributed to wide variation in methodology [23,24].

The potential for caffeine consumption to reduce neuropathology and impart age-associated neuroprotection has motivated numerous studies using animal models. Research using rodent models of neurodegenerative disease generally demonstrates that caffeine administration can successfully alleviate degenerative symptoms and pathology (review by Cunha and Agostinho [25]). Acute treatment with caffeine prevents avoidance memory impairment in a rat model of Parkinson's disease [26], while chronic caffeine treatment prevents cognitive defects in mice expressing toxic forms of amyloid beta, a common rodent model of Alzheimer’s disease [27]. Furthermore, acute caffeine treatment reduces amyloid beta levels in the plasma and brain interstitial fluid [28] and delays memory defects following intracerebral administration of amyloid beta [29-31]. One study using these mice suggests that caffeine treatment may even restore performance in individuals that are already displaying memory deficits [32]. Neurodegenerative disorders aside, caffeine treatment also prevents memory impairment in rodent models of various other diseases and conditions, including chronic stress, child convulsions, type 1 and type 2 diabetes, attention deficit and hyperactivity disorder, heavy alcohol consumption, and sleep deprivation [25]. Importantly, caffeine improves age-associated memory impairment in both mice and rats [33,34].

Recently, several groups have started investigating the physiological effects of caffeine in non-mammalian models, including worms and yeast. A common strategy to model Alzheimer’s disease in *Caenorhabditis elegans* involves transgenic expression of a toxic form of amyloid beta in the body wall muscle, resulting in an age-dependent paralysis phenotype [35]. Dostal et al. [36] found that both caffeine and non-caffeine components of coffee were capable of delaying paralysis in this model. During the course of the present study, another group identified caffeine in a screen for FDA-approved compounds capable of extending worm life span [37]. In yeast, caffeine increases chronological life span, likely through a mechanism related to TOR signaling [38].

Given the high consumption rates of caffeine for people living in the developed world, understanding the impact of caffeine on aging and age-related disease will be important as aging interventions move from the laboratory into use in clinical trials and the broader population. In this study, we characterize the effect of caffeine on life span and healthspan in *C. elegans* and identify clear epistatic interactions between caffeine and both dietary restriction and reduced IIS.

## Results

### Caffeine extends worm life span in a temperature-dependent manner

**Figure 1:**
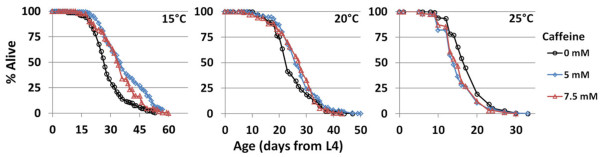
**Caffeine treatment results in a temperature-dependent life span extension in worms.** Maintenance of worms in the presence of 5 mM or 7.5 mM caffeine throughout adult life increases life span at 15°C and 20°C, but not at 25°C.

In order to determine whether caffeine impacts longevity, life span was measured for worms maintained throughout their adult life on nematode growth medium (NGM) plates containing caffeine. In previous work, we observed temperature-dependent effects on life span resulting from reduced expression of the hypoxia-inducible factor, *hif-1*[39]. Specifically, *hif-1* knockdown extended life span at 25°C, but not at 15°C or 20°C. To examine the possibility that caffeine might display a similar dependence on temperature, we measured life span for worms at 15°C, 20°C, and 25°C in the presence of 0 mM, 5 mM, or 7.5 mM caffeine. Caffeine concentrations were selected based on previous studies in yeast and worms [36,38,40,41]. In contrast to *hif-1* knockdown, caffeine extended life span at 15°C and 20°C, and slightly shortened life span at 25°C (Figure 1; Additional file 1: Table S1). These data are in agreement with the recently published FDA-approved drug screen that reported 29.4% median life span extension resulting from 0.1% (5.15 mM) caffeine at 20°C [37]. 

**Figure 2:**
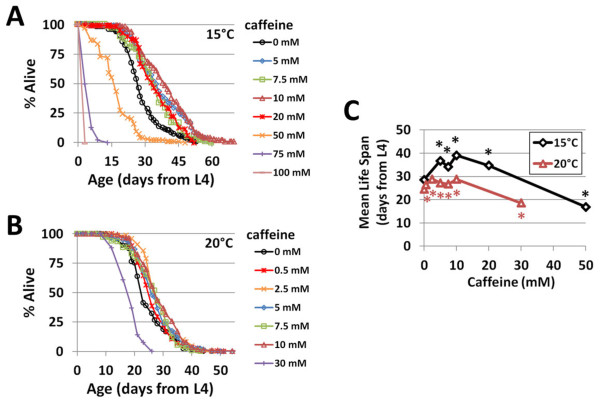
**Caffeine extends worm life span at 15°C and 20°C.** (**A**) Caffeine concentrations in the 5 to 20 mM range increase life span at 15°C. (**B**) Caffeine concentrations in the 0.5 to 10 mM range increase life span at 20°C. (**C**) Caffeine dose response curves reveal optimal concentrations for increased life span. *P < 0.05 vs. 0 mM at the same temperature.

Next, we conducted a caffeine dose response with respect to life span in order to determine the optimal temperature and caffeine concentration for increasing longevity. Life span extension was observed for caffeine concentrations ranging from 0.5 mM to 10 mM at 20°C and from 5 mM to 20 mM at 15°C (Figure 2; Additional file 1: Table S1). The highest mean life span extension (36.7%) was achieved at 15°C using 10 mM caffeine (Figures 2A,C; Additional file 1: Table S1). The highest mean life span extension (16.9%) at 20°C also occurred at 10 mM (Figure 2B,C; Additional file 1: Table S1). Caffeine reduced life span at concentrations of 30 mM or greater at both temperatures (Figure 2; Additional file 1: Table S1).

### Caffeine prolongs mobility and delays polyglutamine-associated pathology

**Figure 3:**
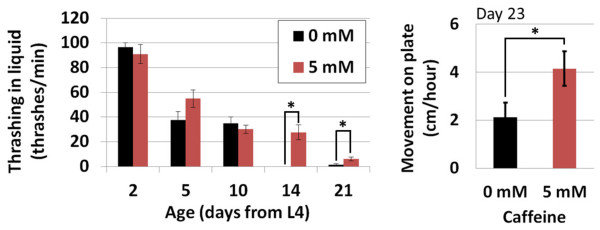
**Caffeine delays age-associated decline in mobility at 15°C.** Worms in the presence of 5 mM caffeine displayed increased thrashing (**A**) and travel rate (**B**) compared to untreated control worms. Error bars represent standard error. *P < 0.05 vs. 0 mM on the same day.

An intervention that increases longevity does not necessarily extend healthspan, the time period over which an organism remains healthy. To assess the effect of caffeine on *C. elegans* healthspan, we examined two types of movement throughout the life span of worms exposed to either 0 mM or 5 mM caffeine. Caffeine delayed age-associated decline in both the thrashing rate in liquid and the rate of travel on solid media in the presence of a bacterial food source (Figure 3).

**Figure 4:**
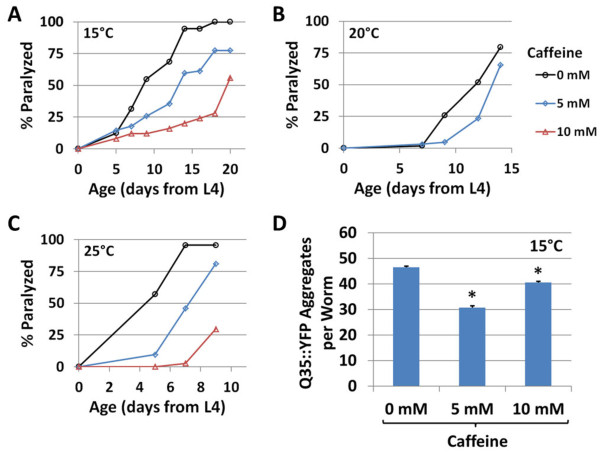
**Caffeine delays age-associated paralysis in a worm polyglutamine toxicity model.** Caffeine treatment delayed the onset of paralysis in worms expressing Q35::YFP in their body wall muscles at (**A**) 15°C, (**B**) 20°C, and (**C**) 25°C. (**D**) Caffeine reduced the formation of Q35::YFP aggregates at 15°C. Error bars represent standard error. *P < 0.05 vs. 0 mM.

Two previous studies found that caffeine can prolong survival [37] and delay the onset of paralysis [36] in a worm model of Alzheimer’s disease in which amyloid beta is express in the body wall muscles [42]. Dostal et al. [36] used an inducible amyloid beta construct that resulted in a severe proteotoxic pathology causing untreated worms to become paralyzed over the course of 20 to 30 h at 20°C. In order to examine the effect of caffeine in a similar model of age-associated proteotoxicity, we examined paralysis in worms expressing an aggregate-prone, YFP-tagged polyglutamine chain (Q35::YFP) in the body wall muscles. Expanded polyglutamine tracts are a known causative factor in Huntington’s disease and related neurodegenerative disorders in humans, though the underlying disease mechanism is not known [43]. Q35::YFP worms display a similar—but more slowly progressing—paralysis phenotype to the amyloid beta worms, with the majority of worms becoming paralyzed over the course of approximately 3 weeks. Similar to the results of the amyloid beta study, we found that caffeine delayed the onset of paralysis in Q35::YFP worms at both 15°C and 20°C (Figure 4A,B). Caffeine also delayed the onset of paralysis at 25°C (Figure 4C), despite the lack of life span extension (Figure 1), suggesting that caffeine may influence life span and proteotoxicity through at least partially distinct mechanisms. We further examined Q35::YFP aggregate formation as a secondary marker of polyglutamine toxicity. Similar to the case for paralysis, caffeine reduced the number of aggregates formed during early adulthood (Figure 4D). 

### Life span extension by bacterial deprivation and exposure to caffeine are non-additive

**Figure 5:**
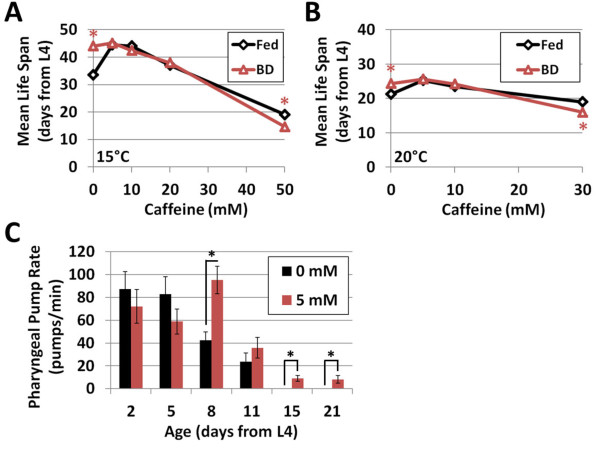
**Caffeine increases life span in a manner that is non-additive with dietary restriction.** Life span extension from caffeine and bacterial deprivation are non-additive at (**A**) 15°C and (**B**) 20°C; *P < 0.05 vs. *ad libitum* fed worms at the same caffeine concentration. (**C**) 5 mM caffeine does not reduce pharyngeal pumping at 15°C. Error bars represent standard error. *P < 0.05 vs. 0 mM on the same day.

Dietary restriction is the most widely studied intervention capable of increasing life span. One goal of dietary restriction research is to discover dietary restriction mimetics, pharmacological agents capable of reproducing the beneficial effects of dietary restriction without a reduction in food intake [44,45]. In order to investigate the potential for caffeine to act as a dietary restriction mimetic, we measured life span for worms with combined exposure to both caffeine and bacterial deprivation, a form of dietary restriction in which the bacterial food source is completely removed after the worms have reached early adulthood [46-48]. Both caffeine and bacterial deprivation extended life span when applied to worms independently, but did not produce an additive increase when combined at either 15°C and 20°C (Figure 5A,B; Additional file 1: Table S1). In fact, bacterial deprivation became detrimental to life span when worms were subjected to caffeine concentrations of 30 mM or greater (Figure 5A,B; Additional file 1: Table S1). The abrogation of life span extension resulting from bacterial deprivation in the presence of caffeine is consistent with the idea that caffeine and dietary restriction influence aging via similar downstream mechanisms. Importantly, caffeine does not mimic dietary restriction by limiting food intake via reduced pharyngeal pumping (Figure 5C), as is the case with long-lived *eat-2* mutants [49,50]. 

### Caffeine extends life span in the absence of *sir-2.1*, *hif-1*, and *cep-1*

**Figure 6:**
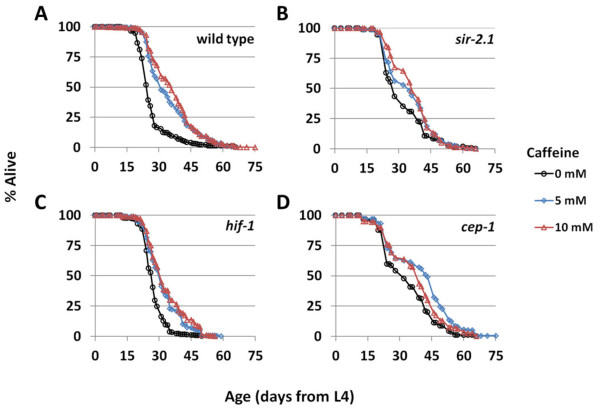
**Caffeine extends life span of canonical longevity mutants.** Treatment with 5 mM or 10 mM caffeine extends life span of (**A**) wild-type, (**B**) *sir-2.1(ok434)*, (**C**) *hif-1(ia4)*, and (**D**) *cep-1(gk138)* worms.

Several genetic pathways are known to influence longevity and may mediate the life span-extending effects of caffeine. In order to identify interactions between caffeine and canonical aging pathways, we measured the effect of caffeine on life span for strains with loss of function mutations in (1) *sir-2.1*, which encodes the worm ortholog of Sir2, a histone deacetylase linked to aging in many species; (2) *hif-1*, which encodes the hypoxia-inducible factor; or (3) *cep-1*, the worm ortholog of the tumor suppressor p53. Based on prior studies showing that bacterial deprivation can increase the life span of worms mutated for either *sir-2.1* or *hif-1*[46,48,51], we predicted that caffeine would also increase life span in these backgrounds. Consistent with this prediction, caffeine increased the life span of *sir-2.1(ok434)* and *hif-1(ia4)* animals (Figure 6A,B,D; Additional file 1: Table S1). Caffeine also increased the life span of *cep-1(gk138)* animals (Figure 6A,C; Additional file 1: Table S1), indicating that the action of caffeine on life span is at least partially independent of all three of these genes, although the magnitude of effect of caffeine on life span in each of these mutant backgrounds was smaller than that observed in wild-type animals at the same concentrations. 

### Caffeine appears to modulate longevity by a mechanism similar to reduced IIS signaling

**Figure 7:**
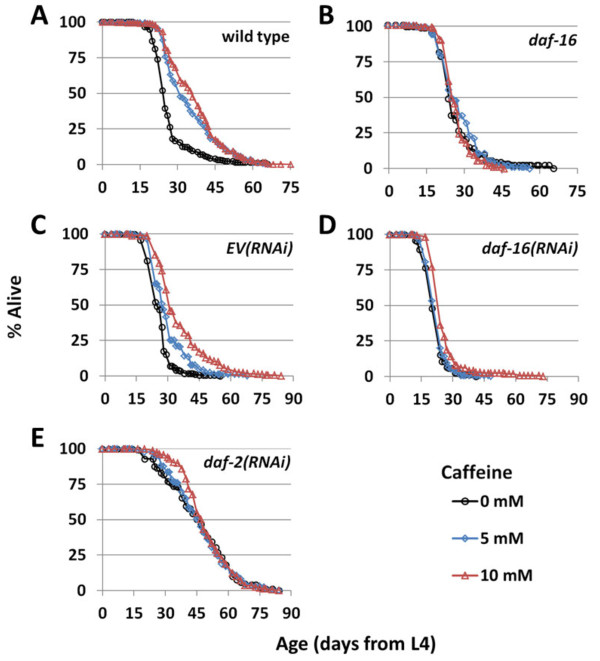
**Caffeine displays epistatic interaction with IIS components.** 5 mM or 10 mM caffeine extends life span in wild-type **(A),** but not *daf-16(mu86) ***(B),** worms, and in wild-type worms fed *EV(RNAi) ***(C)**, but not worms fed *daf-16(RNAi)***(D)** or *daf-2(RNAi) ***(E).**

The IIS pathway is the most widely studied longevity pathway in *C. elegans*. Interventions or mutations that reduce IIS, such as RNAi knockdown of the insulin-like receptor *DAF-2*, increase life span by activating the FOXO family transcription factor DAF-16 [2]. Treatment with 5 mM caffeine failed to extended life span of *daf-16(mu86)* mutant worms (Figure 7A,B; Additional file 1: Table S1). This result is in agreement with a similar finding by Lublin et al. [37] at 20°C and suggests a functional link between caffeine and IIS. To further explore the link between caffeine and IIS, we investigated whether caffeine was capable of extending life span in worms when *daf-16* or *daf-2* was knocked down using RNAi. As with mutation of *daf-16*, 5 mM caffeine failed to increase life span in worms subjected to *daf-16(RNAi)* (Figure 7C,D; Additional file 1: Table S1). In both the case of the *daf-16(mu86)* mutant and the case of wild-type worms subjected to *daf-16(RNAi),* 10 mM caffeine increased life span, though to a much smaller degree than in a wild-type background or in worms subjected to *EV(RNAi)*. This may suggest that a small mechanistic component of the life span extension observed in the presence of caffeine is independent from IIS. Neither 5 mM nor 10 mM caffeine further increased the long life span of worms subjected to *daf-2(RNAi)* (Figure 7C,E; Additional file 1: Table S1). 

**Figure 8:**
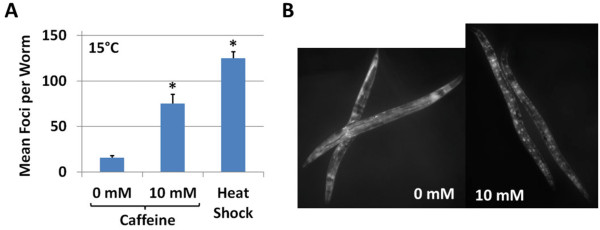
**Caffeine causes DAF-16 nuclear localization.** (**A**) Treatment with 5 mM caffeine causes nuclear localization of transgenically expressed DAF-16::GFP. A 2-h heat shock at 37°C robustly activates DAF-16 and was used as a positive control. Error bars represent standard error. *P < 0.05 vs. 0 mM. (**B**) Representative image showing DAF-16::GFP nuclear localization in response to caffeine.

Activity of DAF-16 is post-translationally regulated through subcellular localization. Reduction of IIS causes dephosphorylation of the DAF-16 protein, allowing it to enter the nucleus and thereby activate transcription of target genes. In order to determine whether caffeine influences DAF-16 localization in a this manner, we examined transgenic worms expressing a GFP-tagged DAF-16 protein (DAF-16::GFP). Worms exposed to caffeine for 2–3 h displayed an increase in DAF-16::GFP nuclear localization compared to untreated controls (Figure 8). These data are consistent with a model in which caffeine impacts life span, at least in part, by reducing insulin signaling and activating DAF-16.

**Figure 9:**
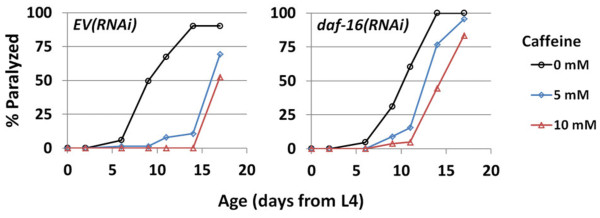
**Caffeine delays age-associated paralysis associated with polyglutamine toxicity independently from DAF-16.** Caffeine treatment delayed the onset of paralysis in worms expressing Q35::YFP in their body wall subject to either *EV(RNAi)* or *daf-16(RNAi)* at 15°C.

We next asked whether DAF-16 is necessary for caffeine to delay paralysis in the Q35::YFP polyglutamine toxicity model. Intriguingly, caffeine was capable of delaying paralysis of the Q35::YFP worms even in the presence of *daf-16(RNAi)* (Figure 9), adding further support for a model in which caffeine impacts life span and polyglutamine toxicity through at least partially distinct mechanisms.

## Discussion

In this study, we demonstrate that chronic exposure to caffeine during adulthood extends life span and healthspan of *C. elegans* in a temperature-dependent manner. Life span extension from caffeine is non-additive with life span extension by bacterial deprivation and independent of the hypoxia inducible factor, HIF-1, the *C. elegans* p53 ortholog, CEP-1, and the *C. elegans* Sir2 ortholog, SIR-2.1. Caffeine appears to act, at least in part, by activating the FOXO transcription factor DAF-16 in a manner similar to reduced IIS. Chronic caffeine exposure also delays paralysis in a *C. elegans* model of polyglutamine disease, though this effect appears to be independent of DAF-16.

Perhaps the most intriguing therapeutic value for caffeine is its potential to reduce the risk and delay the onset of age-associated neurodegenerative disease. As described in the introduction, studies in humans and rodents indicate that caffeine treatment reduces the risk of disease onset and improves cognitive decline in models of Alzheimer’s and Parkinson’s disease. Previous work has also shown that caffeine is capable of delaying pathology in worm models of Alzheimer’s disease [36,37]. In this study, we expand upon these findings to show that caffeine is capable of delaying pathology in a worm model of polyglutamine disease. Although mammalian studies investigating the use of caffeine in neurodegenerative disorders have focused primarily on Alzheimer’s and Parkinson’s disease, research that examines the effects of caffeine consumption on Huntington’s disease in human populations is currently underway. Our finding suggests that a more detailed examination of the influence of caffeine on the progression of a broader spectrum of diseases associated with proteotoxic stress in mammalian models may be warranted.

This study and that by Lublin et al. [37] both identify an epistatic interaction between caffeine and IIS. A related link has been made in mammals. While acute treatment with caffeine has been shown to increase blood pressure [52] and reduce insulin sensitivity [53,54], long-term coffee consumption shows a strong correlation with reduced risk of type 2 diabetes in humans [55], and chronic caffeine exposure prevents diet-induced insulin resistance and hypertension in rats [56]. Chronic caffeine consumption may prove useful in mimicking reduced IIS and improving diet-induced insulin resistance.

The interaction observed between bacterial deprivation and caffeine suggests that caffeine may also emulate aspects of dietary restriction, which is particularly interesting given that caffeine is already in common use in human society. The observation that caffeine interacts with both bacterial deprivation and IIS is complicated by the fact that bacterial deprivation extends life span independently of both DAF-2 and DAF-16 [46,48], suggesting that caffeine may activate overlapping downstream targets in both pathways. The additional observation that life span extension from caffeine is reduced in three other strain backgrounds with mutations in genes linked to aging suggests that caffeine may be activating a common set of cellular processes important for increased longevity via a range of interventions. The idea that caffeine may interact with multiple pathways involved in aging is further supported by the observation that the influences of caffeine on life span and polyglutamine toxicity are separable with respect to their dependence on both temperature and DAF-16. Alternatively, it is possible that caffeine alters the molecular or physiological state of the organisms in such a way that renders the organism unable to respond normally to signals that result in increased life span in untreated animals. Epistatic interactions provide limited information regarding the potential mechanism of life span extension [57], and further study will be required to unravel the complexities of caffeine’s impact on the aging process.

This study identifies genetic interaction between caffeine and both the IIS and the dietary restriction pathways. The direct molecular targets and downstream mechanisms by which caffeine influences longevity remain to be investigated. Mammalian research suggests that caffeine primarily impacts cognitive phenotypes by antagonizing adenosine receptors A_1_ and A_2A_[25]. At sub-toxic levels of caffeine, adenosine receptors are the only clear molecular targets and many of the beneficial effects of caffeine are mimicked by specific agonists of adenosine receptor A_2A_[25]. Mild inhibition of phosphodiesterase activity has only been observed at higher concentrations of caffeine [58]. Functionally, caffeine prevents memory impairment induced by heavy alcohol consumption in rats, an effect that can be mimicked by simultaneous treatment with inhibitors of phosphodiesterase 5 and adenosine receptor A_2A_ (but not by either inhibitor alone) [58]. This suggests that specific subclasses of phosphodiesterases may be important for the action of sub-toxic doses of caffeine in some circumstances.

Clear orthologs of mammalian adenosine receptors have not yet been identified in *C. elegans*, though there are several candidate genes based on sequence homology. In contrast, the *C. elegans* genome contains six phosphodiesterases that fall into two functional classes: one class that specifically targets cAMP (PDE-4,6) and another that is thought to target cGMP (PDE-1,2,3,5) [59,60]. High doses of caffeine have been shown to inhibit mammalian cAMP phosphodiesterases [61,62], indicating that the former class is of greater interest. Further investigation will determine whether adenosine receptors, phosphodiesterases, or other targets are involved in caffeine’s effect on worm life span.

Two factors have complicated mammalian research with caffeine. In human populations, most studies determine caffeine intake by consumption of caffeine-containing foods and beverages, such as coffee, tea, soft drinks, and chocolate, all of which contain other compounds that have the potential to affect the diseases under investigation. For example, one study found that long-term coffee consumption shows a strong correlation with reduced risk of type 2 diabetes [55], while a later study found a similar correlation for both caffeinated and decaffeinated coffee consumption [63]. A recently published study identified a correlation between consumption of either caffeinated or decaffeinated coffee and reduced mortality risk for a range of age-associated diseases, including diabetes [64]. The collective findings from these studies suggest that some of the insulin-related benefit from coffee consumption may result from compounds in coffee other than caffeine. Interpretations are further complicated by seemingly contradictory effects resulting from acute and chronic caffeine treatment in some circumstances, as discussed previously with respect to insulin sensitivity and hypertension. *C. elegans* may be a useful model for decoupling these types of complications. For example, worm studies completed to date have already begun to separate influences from caffeine and non-caffeine sources with respect to coffee. Dostal et al. [36] identified SKN-1 as a primary downstream factor in the caffeine-independent delay in amyloid beta toxicity using coffee extract, while this study and that by Lublin et al. [37] identify IIS as an important player in life span extension by caffeine.

A growing accumulation of evidence in humans, rodents, and nematodes suggests that chronic caffeine exposure may yield significant health benefits by delaying aging and preventing specific age-associated pathologies. Recent studies indicate that *C. elegans* will be a useful system for dismantling the molecular events that underlie the beneficial effects of caffeine. *C. elegans* research may also help unravel the complications associated with acute versus chronic caffeine treatment and identify other compounds in coffee and tea with the potential to promote longer life span. Overall, based on the observations that caffeine is capable of increasing life span in worms and has been correlated with decreased mortality in humans, we anticipate the expansion of studies examining the influence of caffeine on longevity into mammalian systems.

## Conclusion

In this study, we have validated and extended prior evidence that caffeine can improve healthy aging in *C. elegans* by showing that caffeine can both increase life span and promote resistance to proteotoxic stress. The longevity effects of caffeine appear to overlap with both the insulin-like signaling pathway and with dietary restriction, while the resistance to polyglutamine toxicity is independent of insulin-like signaling. The effects of caffeine on longevity also vary based on the experimental temperature. It will be important to unravel the mechanisms underlying these observations in future studies. In this way, it may be possible to define how caffeine exerts its positive health benefits in worms and determine whether similar processes govern the effects of caffeine on human longevity and healthspan.

## Methods

### Strains and media

The strains used in this study are listed in Additional file 1: Table S2 and were obtained from the *Caenorhabditis* Genetics Center, the laboratory of Dr. Chris Link (University of Colorado, Boulder, CO, USA), or the laboratory of Dr. Jim Thomas (University of Washington, Seattle, WA, USA). Animals were maintained on solid nematode growth media (NGM) agar plates using standard techniques. Experiments were performed on NGM plates supplemented with 25 mg/ml ampicillin to prevent contamination. Adult worms were placed on NGM plates containing FUdR to prevent reproduction. With the exception of the bacteria used in RNAi experiments, bacterial food was killed by exposure to UV and addition of ampicillin to the medium, as previously described [65]. Solid anhydrous caffeine (MP Biomedicals, Solon, OH, USA) was added directly to the NGM solution prior to autoclaving. Neither autoclaving nor UV treatment during plate preparation influenced life span in the presence of caffeine (data not shown). All experiments were conducted at 15°C except where otherwise noted.

### RNAi

RNAi experiments were conducted using feeding protocols according to standard procedures. The RNAi feeding strains targeting *daf-16* and *daf-2* were obtained from the Vidal RNAi library [66] and J. McElwee, respectively. RNAi plasmids were sequenced to verify the target sequence. RNAi plates consisted of NGM supplemented with 1 mM β-D-isothiogalactopyranoside (IPTG) and 25 μg/ml carbenicillin. Worms were raised on RNAi bacteria from egg to the L4 stage of development and then transferred to plates containing freshly seeded RNAi bacteria plus 50 μM FUdR to prevent production.

### Life span analysis

Life span experiments were conducted as previously described [65]. Bacterial deprivation was conducted by maintaining animals on UV-killed OP50 bacteria food until day 4 of adulthood and then transferring animals to NGM agar plates containing ampicillin but without a bacterial food source, as previously described [47,67].

### Paralysis analysis

Paralysis of worms was assessed visually as previously described [68]. Worms were scored as paralyzed if they were unable to make forward progress on the NGM surface in response to plate-tapping or tail-prodding.

### Quantification of aggregates

Q35::YFP aggregates were quantified by taking images using fluorescent microscopy followed by automated quantification of the number of aggregates present per worm based on pixel density using ImageJ.

### Movement assays

Thrashing was quantified visually by suspending individual animals in a droplet of M9 buffer on the surface of an NGM plate and counting the number of body bends in 60 s. Movement rate was quantified by placing an individual worm onto a fresh OP50-seeded NGM plate. After 60 min, pictures were taken of tracks left in the bacterial lawn using a standard SLR camera with a microscope eyepiece adapter. Track length was measured using ImageJ software. Pharyngeal pumping was quantified visually by taking video recordings of the head region of individual animals and counting the number of pumps in 30 s.

### DAF-16::GFP nuclear localization

Transgenic worms expressing DAF-16::GFP were transferred to plates containing 0 mM or 5 mM caffeine at the L4 stage of development for. A third population of worms was exposed to 30°C for 2 h immediately prior to analysis. Worms were immobilized by treatment with 25 nM NaN_3_ and still images captured using a mounted digital camera and the GFP fluorescence channel of a Zeiss SteREO Lumar V.12 microscope. Captured images were used to quantify visible GFP foci.

### Statistical analysis

Statistical significance was determined for worm life span using the Wilcoxon rank sum test. All other comparisons were conducted using an unpaired two-tailed Student’s *T*-test assuming unequal variance. 

## Competing interests

The authors declare that they have no competing interests.

## Authors’ contributions

GLS conceived of this study, coordinated experiments, carried out life span assays, and drafted the manuscript. EB carried out life span, movement, pharyngeal pumping, and DAF-16::GFP nuclear localization assays. MEY and RMM carried out paralysis and aggregate quantification assays. MK provided oversight for experimental design and coordination, and helped draft the manuscript. All authors read the manuscript, provided constructive commentary and criticism, and approved final submission.

## Supplementary Material

Additional file 1 **Table S1.** Summary of life span data in this study. **Table S2.** Strains used in this study. Click here for file
