# Supplementary material for: Caffeine extends life span, improves healthspan, and delays age-associated pathology in Caenorhabditis elegans
Source: Longev Healthspan. 2012 Dec 1;1:9. doi: 10.1186/2046-2395-1-9 (PMC3922918; doi:10.1186/2046-2395-1-9)
Supplement: Additional file 1 — Table S1. Summary of life span data in this study. Table S2. Strains used in this study. [file 2046-2395-1-9-S1.pdf]

Table S1

	5 mM caffeine BD	20°C	26.0	25.6	73	0 mM caffeine BD	20°C	23.0	24.4	61	0.1006	5.3%	no effect
	10 mM caffeine BD	20°C	23.0	24.2	66	0 mM caffeine BD	20°C	23.0	24.4	61	0.9981	-0.5%	no effect
	30 mM caffeine BD	20°C	4.0	16.0	16	0 mM caffeine BD	20°C	23.0	24.4	61	7.43E-19	-34.3%	decreased
IIS RNAi	*EV(RNAi)* 5 mM caffeine	15°C	28.0	30.2	213	*EV(RNAi)* 0 mM caffeine	15°C	25.0	25.9	253	8.62E-09	16.6%	increased
(Figure 7C,D,E)	*EV(RNAi)* 10 mM caffeine	15°C	31.0	35.7	195	*EV(RNAi)* 0 mM caffeine	15°C	25.0	25.9	253	5.64E-28	37.6%	increased
	daf-2(RNAi) 0 mM caffeine	15°C	47.0	45.6	218	*EV(RNAi)* 0 mM caffeine	15°C	25.0	25.9	253	4.30E-46	75.7%	increased
	daf-2(RNAi) 5 mM caffeine	15°C	46.0	46.4	266	*EV(RNAi)* 5 mM caffeine	15°C	28.0	30.2	213	1.80E-40	53.5%	increased
	daf-2(RNAi) 10 mM caffeine	15°C	47.5	49.1	206	*EV(RNAi)* 10 mM caffeine	15°C	31.0	35.7	195	1.40E-26	37.6%	increased
	daf-2(RNAi) 5 mM caffeine	15°C	46.0	46.4	266	*daf-2(RNAi)* 0 mM caffeine	15°C	47.0	45.6	218	0.6015	1.9%	no effect
	daf-2(RNAi) 10 mM caffeine	15°C	47.5	49.1	206	*daf-2(RNAi)* 0 mM caffeine	15°C	47.0	45.6	218	0.0148	7.7%	increased
	daf-16(RNAi) 0 mM caffeine	15°C	20.0	21.5	215	*EV(RNAi)* 0 mM caffeine	15°C	25.0	25.9	253	1.79E-20	-16.9%	decreased
	daf-16(RNAi) 5 mM caffeine	15°C	24.0	22.3	272	*EV(RNAi)* 5 mM caffeine	15°C	28.0	30.2	213	6.61E-35	-26.2%	decreased
	daf-16(RNAi) 10 mM caffeine	15°C	24.0	25.8	180	*EV(RNAi)* 10 mM caffeine	15°C	31.0	35.7	195	9.79E-28	-27.7%	decreased
	daf-16(RNAi) 5 mM caffeine	15°C	24.0	22.3	272	*daf-16(RNAi)* 0 mM caffeine	15°C	20.0	21.5	215	0.0884	3.6%	no effect
	daf-16(RNAi) 10 mM caffeine	15°C	24.0	25.8	180	*daf-16(RNAi)* 0 mM caffeine	15°C	20.0	21.5	215	4.51E-12	19.7%	increased
Longevity Pathway	5 mM caffeine	15°C	31.0	34.6	419	0 mM caffeine	15°C	25.0	26.6	416	1.25E-38	29.8%	increased
Epistasis	10 mM caffeine	15°C	36.0	36.5	426	0 mM caffeine	15°C	25.0	26.6	416	3.10E-54	37.1%	increased
(Figure 6;7A,B)	*daf-16* 0 mM caffeine	15°C	24.0	26.8	243	0 mM caffeine	15°C	25.0	26.6	416	0.8121	0.7%	no effect
	daf-16 5 mM caffeine	15°C	25.5	27.8	216	5 mM caffeine	15°C	31.0	34.6	419	4.04E-16	-19.7%	decreased
	daf-16 10 mM caffeine	15°C	25.5	26.9	242	10 mM caffeine	15°C	36.0	36.5	426	1.06E-36	-26.3%	decreased
	daf-16 5 mM caffeine	15°C	25.5	27.8	216	*daf-16* 0 mM caffeine	15°C	24.0	26.8	243	0.0613	3.5%	no effect
	daf-16 10 mM caffeine	15°C	25.5	26.9	242	*daf-16* 0 mM caffeine	15°C	24.0	26.8	243	0.0196	0.4%	increased
	cep-1 0 mM caffeine	15°C	32.0	33.5	186	0 mM caffeine	15°C	25.0	26.6	416	3.77E-10	25.8%	increased
	cep-1 5 mM caffeine	15°C	43.0	39.8	177	5 mM caffeine	15°C	31.0	34.6	419	0.0003	15.3%	increased
	cep-1 10 mM caffeine	15°C	39.0	37.2	160	10 mM caffeine	15°C	36.0	36.5	426	0.5192	1.8%	no effect
	cep-1 5 mM caffeine	15°C	43.0	39.8	177	*cep-1* 0 mM caffeine	15°C	32.0	33.5	186	2.81E-06	18.9%	increased
	cep-1 10 mM caffeine	15°C	39.0	37.2	160	*cep-1* 0 mM caffeine	15°C	32.0	33.5	186	0.0033	11.0%	increased
	sir-2.1 0 mM caffeine	15°C	28.0	31.9	159	0 mM caffeine	15°C	25.0	26.6	416	3.05E-09	19.7%	increased
	sir-2.1 5 mM caffeine	15°C	35.0	34.8	181	5 mM caffeine	15°C	31.0	34.6	419	0.9844	0.8%	no effect
	sir-2.1 10 mM caffeine	15°C	36.0	36.4	158	10 mM caffeine	15°C	36.0	36.5	426	0.8455	-0.3%	no effect
	sir-2.1 5 mM caffeine	15°C	35.0	34.8	181	*sir-2.1* 0 mM caffeine	15°C	28.0	31.9	159	0.0055	9.3%	increased
	sir-2.1 10 mM caffeine	15°C	36.0	36.4	158	*sir-2.1* 0 mM caffeine	15°C	28.0	31.9	159	3.17E-06	14.2%	increased
	hif-1 0 mM caffeine	15°C	27.0	27.2	404	0 mM caffeine	15°C	25.0	26.6	416	1.09E-07	2.0%	increased
	hif-1 5 mM caffeine	15°C	31.0	31.6	340	5 mM caffeine	15°C	31.0	34.6	419	0.0023	-8.6%	decreased
	hif-1 10 mM caffeine	15°C	31.0	33.0	294	10 mM caffeine	15°C	36.0	36.5	426	2.06E-05	-9.7%	decreased
	hif-1 5 mM caffeine	15°C	31.0	31.6	340	*hif-1* 0 mM caffeine	15°C	27.0	27.2	404	2.92E-14	16.2%	increased
	hif-1 10 mM caffeine	15°C	31.0	33.0	294	*hif-1* 0 mM caffeine	15°C	27.0	27.2	404	7.95E-20	21.3%	increased

	Intervention					Control							
Experiment	Name	Temp.	Median Life Span	Mean Life Span	N	Name	Temp.	Median Life Span	Mean Life Span	N	Rank Sum P-Value	Change in Mean Life	Effect on Life Span
Dose response	5 mM caffeine	15°C	35.0	36.7	977	0 mM caffeine	15°C	27.0	28.6	1037	1.84E-83	28.5%	increased
(Figures 1;2)	7.5 mM caffeine	15°C	34.0	34.2	186	0 mM caffeine	15°C	27.0	28.6	1037	8.92E-20	19.6%	increased
	10 mM caffeine	15°C	39.0	39.1	644	0 mM caffeine	15°C	27.0	28.6	1037	2.97E-98	36.7%	increased
	20 mM caffeine	15°C	34.0	34.7	227	0 mM caffeine	15°C	27.0	28.6	1037	3.10E-22	21.6%	increased
	50 mM caffeine	15°C	17.0	16.9	188	0 mM caffeine	15°C	27.0	28.6	1037	3.70E-55	-40.9%	decreased
	75 mM caffeine	15°C	3.0	4.9	57	0 mM caffeine	15°C	27.0	28.6	1037	7.07E-37	-82.8%	decreased
	100 mM caffeine	15°C	3.0	3.0	83	0 mM caffeine	15°C	27.0	28.6	1037	5.02E-52	-89.5%	decreased
	0.5 mM caffeine	20°C	26.0	26.5	186	0 mM caffeine	20°C	23.0	24.6	575	0.0001	7.7%	increased
	2.5 mM caffeine	20°C	28.0	28.8	167	0 mM caffeine	20°C	23.0	24.6	575	1.12E-15	17.2%	increased
	5 mM caffeine	20°C	26.0	27.3	523	0 mM caffeine	20°C	23.0	24.6	575	8.27E-13	10.8%	increased
	7.5 mM caffeine	20°C	28.0	26.7	243	0 mM caffeine	20°C	23.0	24.6	575	8.54E-08	8.7%	increased
	10 mM caffeine	20°C	28.0	28.8	227	0 mM caffeine	20°C	23.0	24.6	575	1.87E-14	16.9%	increased
	30 mM caffeine	20°C	19.0	18.6	92	0 mM caffeine	20°C	23.0	24.6	575	1.55E-18	-24.4%	decreased
	5 mM caffeine	25°C	14.0	15.4	178	0 mM caffeine	25°C	17.0	17.8	170	1.97E-07	-13.6%	decreased
	7.5 mM caffeine	25°C	15.0	15.8	165	0 mM caffeine	25°C	17.0	17.8	170	2.54E-05	-11.6%	decreased
Bacteria Deprivation	0 mM caffeine BD	15°C	48.0	44.0	136	0 mM caffeine	15°C	31.0	33.6	248	1.57E-16	31.2%	increased
(Figure 5A,B)	5 mM caffeine BD	15°C	45.0	45.1	131	5 mM caffeine	15°C	48.0	44.5	228	0.8533	1.5%	no effect
	10 mM caffeine BD	15°C	43.0	42.4	146	10 mM caffeine	15°C	47.0	44.0	218	0.0764	-3.7%	no effect
	20 mM caffeine BD	15°C	38.0	37.9	58	20 mM caffeine	15°C	38.0	37.1	159	0.5511	2.4%	no effect
	50 mM caffeine BD	15°C	14.0	14.7	95	50 mM caffeine	15°C	19.0	19.1	141	4.47E-08	-23.1%	decreased
	5 mM caffeine	15°C	48.0	44.5	228	0 mM caffeine	15°C	31.0	33.6	248	3.09E-29	32.5%	increased
	10 mM caffeine	15°C	47.0	44.0	218	0 mM caffeine	15°C	31.0	33.6	248	1.90E-24	31.1%	increased
	20 mM caffeine	15°C	38.0	37.1	159	0 mM caffeine	15°C	31.0	33.6	248	0.0004	10.4%	increased
	50 mM caffeine	15°C	19.0	19.1	141	0 mM caffeine	15°C	31.0	33.6	248	2.82E-42	-43.0%	decreased
	5 mM caffeine BD	15°C	45.0	45.1	131	0 mM caffeine BD	15°C	48.0	44.0	136	0.9406	2.5%	no effect
	10 mM caffeine BD	15°C	43.0	42.4	146	0 mM caffeine BD	15°C	48.0	44.0	136	0.0649	-3.8%	no effect
	20 mM caffeine BD	15°C	38.0	37.9	58	0 mM caffeine BD	15°C	48.0	44.0	136	0.0001	-13.9%	decreased
	50 mM caffeine BD	15°C	14.0	14.7	95	0 mM caffeine BD	15°C	48.0	44.0	136	3.79E-35	-66.6%	decreased
	0 mM caffeine BD	20°C	23.0	24.4	61	0 mM caffeine	20°C	21.0	21.3	70	0.0001	14.4%	increased
	5 mM caffeine BD	20°C	26.0	25.6	73	5 mM caffeine	20°C	26.0	25.3	92	0.5703	1.4%	no effect
	10 mM caffeine BD	20°C	23.0	24.2	66	10 mM caffeine	20°C	23.0	23.5	72	0.1699	3.1%	no effect
	30 mM caffeine BD	20°C	4.0	16.0	16	30 mM caffeine	20°C	4.0	19.0	18.6	1.17E-05	-15.8%	decreased
	5 mM caffeine	20°C	26.0	25.3	92	0 mM caffeine	20°C	21.0	21.3	70	2.66E-07	18.8%	increased
	10 mM caffeine	20°C	23.0	23.5	72	0 mM caffeine	20°C	21.0	21.3	70	0.0061	10.4%	increased
	30 mM caffeine	20°C	4.0	19.0	19	0 mM caffeine	20°C	21.0	21.3	70	0.0012	-10.7%	decreased

Table S2

Strain	Genotype
N2	wild type
CF1038	*daf-16(mu86) I*
VC199	*sir-2.1(ok434) IV*
TJ1	*cep-1(gk138) I*
ZG31	*hif-1(ia4) V*
TJ356	zIs356[*pdaf-16::daf-16-gfp; rol-6*]
MQ35	*Q35::YFP*
